# Cross-national Differences in Intergenerational Family Relations: The Influence of Public Policy Arrangements

**DOI:** 10.1093/geroni/igx032

**Published:** 2018-01-04

**Authors:** Pearl A Dykstra

**Affiliations:** Department of Public Administration and Sociology, Erasmus University Rotterdam, The Netherlands

**Keywords:** Intergenerational family relations, Interdependence, Cross-national, Europe, Policy

## Abstract

Focusing mostly on Europe, this overview reveals how the research on cross-national differences in intergenerational family relations has moved from basic descriptions to a focus on understanding how support exchanges are shaped by macro-level processes. A key issue concerns generational interdependence, the extent to which public policy arrangements impose reliance on older and younger family members or enable individual autonomy. Real theoretical progress is visible in three areas of research. The first pertains to analyses at the micro level of how family members actually respond to the incentives that different macro contexts provide. The generosity or restrictedness of public provisions variably releases or necessitates normative obligations in interdependent family relationships. The second area of progress involves analyses of the implications of specific policies rather than policy packages for gender and socioeconomic inequality. The third area of progress is a more nuanced view on the familialism–individualism divide. These three areas provide inspiring examples for future investigations.

**Translational Significance**

Citizens and policy makers will benefit from knowledge about the different implications that different policies have for gender and socioeconomic inequality. Cash for care payments, which are taken more easily by women than men and by low-paid women than high-paid women, increases the likelihood of poverty in advanced age. Care services better assist men and women in reconciling family care and paid work.

In the past 15 years, the literature on cross-national differences in intergenerational family relations has moved from basic descriptions of support exchanges to a focus on understanding how support exchanges are shaped by macro-level processes. The challenge of linking family practices to structural forces is a fertile ground for theoretical development. Much of the scholarship has focused on European multigenerational families due to the availability of large-scale comparative data collections, such as the Survey of Health, Ageing and Retirement in Europe (SHARE), in which 26 European countries and Israel currently participate, and the Generations and Gender Survey (GGS), in which 19 European and 4 non-European countries currently participate. (Countries have joined in different years, so data sets for the full range of countries are not yet available. For more information on the surveys, visit http://www.share-project.org, last accessed November 27, 2017, and http://www.ggp-i.org, last accessed November 27, 2017) In this article, I describe major findings, focusing mostly on Europe, and critically reflect on the conceptual progress that has been made. 

## Weak and Strong Family Regions

Reher’s (1998) article on family ties in western Europe has served as a source of inspiration for many cross-national studies. In “bold strokes” (p. 204), he described the center and north of Europe as a weak family region and the Mediterranean as a strong family region. In countries with weak family ties, young adults set up households of their own at a relatively young age, and the provision of care to vulnerable family members is largely accomplished through public and private institutions. In countries with strong family ties, young adults remain in the parental home until they marry, and much of the aid given to the needy and the poor comes from the family. In weak family areas, individualistic values prevail, whereas collectivistic values predominate in strong family contexts. Reher traced the emphasis on the individual and self-reliance in northern Europe to the Reformation and attributed the overriding importance of kin ties in southern Europe to Catholic and Islamic influences.

The first cross-national studies on intergenerational ties using data from SHARE focused on co-residence, spatial proximity, and frequency of contacts. Results demonstrated “not only a ‘weak’–‘strong’ dichotomy but a North-South gradient” (Kohli, Künemund, & Lüdike, 2005, p. 167). In Scandinavian countries, the proportions of older adults with children in the same household, at least one child living within a 25-km radius, and daily contact with at least one child are lower than in the Mediterranean countries, with the Continental European countries somewhere in the middle (Hank, 2007). Interestingly, country differences in intergenerational transfers of time and money do not clearly fit Reher’s division between weak family and strong family regions (Albertini, Kohli, & Vogel, 2007; Bonsang, 2007; Hank & Buber, 2009; Ogg & Renaut, 2006).

Following Reher, one would expect both the frequency and intensity of intergenerational transfers to be lowest in weak family regions and highest in strong family regions. Results show otherwise. The Scandinavian countries exhibit the highest frequency of giving and receiving, but the lowest intensity of support exchanges. The frequency of support exchanges is lowest in the Mediterranean countries, but the intensity is highest. Again, support transfers in the continental European countries fall in between the other two regions. Clearly, support for Reher’s weak family—strong family dichotomy depends on the measure of intergenerational relations that is used.

Until recently, research on intergenerational relations rarely included Eastern European countries, with the exception of scholarship inspired by Hajnal’s (1965, 1982) dividing line that runs from St. Petersburg, Russia, to Trieste, Italy. Increasingly, data on intergenerational exchanges in Eastern Europe are becoming available. The European Union Statistics on Income and Living Conditions (EU-SILC) report levels of intergenerational coresidence in Eastern Europe that often parallel those in southern Europe (Aassve, Cottini, & Vitatli, 2013; Iacovou & Skew, 2011). Note, however, that the prevalence of coresidential arrangements in the Czech Republic, Hungary, and the Baltic States resembles that in continental Europe, serving as a reminder that researchers should not engage in what Szoltysek (2012) describes as a “Western homogenising view of Eastern European family patterns” (p. 12). Mönkediek and Bras (2014) included the Czech Republic and Poland in their analyses of subnational variations in family structures. They classify these central and Eastern European regions in terms of “rather weak” (p. 252) family regimes: family members tend to live near one another, but levels of contact are relatively low.

## Transfer Regimes

The term “transfer regime” (Albertini et al., 2007) was introduced to interpret the cross-national findings on intergenerational exchanges, thereby stressing the correspondence with established classifications of countries based on the decommodification of public transfers and services (Esping-Andersen, 1990). Two considerations are crucial here. The first is that generous welfare provisions help relieve family and kin from the burden of economic support and personal care. Rather than “crowding out” family care, generous welfare state services actually complement it (Daatland & Lowenstein, 2005; Künemund & Rein, 1999; Motel-Klingebiel, Tesch-Römer, & Von Kondratowitz, 2005). The second consideration is that public transfers might be redistributed at the family level. With regard to downward family support, monetary welfare provisions enable family members to respond to those with the greatest financial needs (Kohli, 1999). Interactions between family and state support merit attention because private transfers have important implications for the labor supply of helpers and recipients as well as their capital accumulation (Attias-Donfut, Ogg, & Wolff, 2005).

Subsequent studies using SHARE data aimed to reveal why intergenerational transfer patterns are correlated with welfare regimes. The typical approach is to connect different kinds of intergenerational assistance to relevant measures of welfare provisions in multilevel models (e.g., Brandt, 2013; Brandt & Deindl, 2013; Deindl & Brandt, 2011; Haberkern & Szydlik, 2010; Igel, Brandt, Haberkern, & Szydlik, 2009; Igel & Szydlik, 2011). Brandt, Haberkern, and Szydlik’s (2009) article is perhaps the most noteworthy on this topic: it reveals that the availability of social service professionals in a given country shapes the types of supportive tasks that adult children perform for their aging parents. The authors distinguished practical help (e.g., assistance with household tasks, paperwork) and physical care (e.g., assistance with bathing, dressing, eating) given to parents and took the size of the social service sector (measured as the percentage of employees in that sector) as indicator of welfare provisions. Findings show that the proportion of adult children providing practical help to parents is higher, but the proportion providing physical care is lower in countries with a larger social service sector. There is a “crowding in” of practical help, but a “crowding out” of physical care. When professionals take on the complex, demanding, and routinizable physical care tasks, family members have greater opportunities to provide spontaneous and nontechnical forms of support. A drawback of the Brandt and colleagues’ study is that the measure of social services conflates publicly and privately funded arrangements. State provisions cannot be distinguished from market provisions.

Another noteworthy study is that of Mudrazija (2016), who shows that public redistribution of resources between parents and children is associated with a secondary redistribution in the opposite direction at the family level. The author focuses on policies affecting intergenerational redistribution, namely public spending on old-age and survivors’ insurance benefits (OASI) and family policy, both measured as share of gross domestic product. His interest is in the net beneficiary (defined as the monetary value of financial and nonfinancial transfers that parents give to children, minus the monetary value of transfers they receive from children) at different stages in life. Across most European countries, children are the net beneficiary of transfers up until the point when parents reach an advanced age (80 and older). Results show furthermore that higher OASI to family spending ratio is associated with larger net transfers from parents to children, suggesting that parents provide more support to adult children or children decrease their support to parents when public intergenerational redistribution of resources becomes relatively more favorable for parents than their children, and vice versa. It is common practice to use social expenditures as welfare regime indicator, as Mudrazija has done. The drawback is, however, that expenditures can cover different policy packages: income transfers or services in kind, so that the effects of specific policies remain unclear. I will return to this point later.

## How Transfer Regimes Shape Generational Interdependence

The literature on intergenerational transfer regimes has made considerable strides toward mapping exchange patterns at the micro level of families to characteristics of welfare states. Concepts such as “specialization” (Igel et al., 2009) and “redistribution” (Mudrazija, 2014) provide telling descriptions of patterned links between public and private streams of intergenerational support. Nevertheless, the research community has only started to scratch the surface of how the macro-level welfare regime context shapes mechanisms of transfers at the micro level of family behavior. A key issue concerns generational interdependence (Dykstra & Hagestad, 2016; Hagestad & Dykstra, 2016): the extent to which public policy arrangements impose reliance on older and younger family members or enable individual autonomy (Leitner, 2003; Saraceno & Keck, 2010; Zagel & Lohmann, 2016). Generational interdependence exists when family members of multiple generations are emotionally, financially, practically, and morally reliant on and responsible to each other. It is a complex phenomenon, in that it has rewarding elements such as rights, support, continuity, and protection against risks, as well as unsettling elements such as obligations, vulnerabilities related to events and resources of others, and transitions beyond a person’s control.

Albertini and Kohli (2013) nicely demonstrate how generational interdependence in the family realm varies, depending on the transfer regime context. They focus on the needs and resources of parents and children as determinants of residential autonomy of the younger generation. For reasons of parsimony, I will focus on the findings for children’s employment status and parental education. In southern and continental European countries, children without a job are less likely than children with a job to live on their own. In Scandinavian countries, however, the likelihood of residential autonomy does not vary across children’s employment statuses. In Sweden and Denmark, higher levels of welfare decommodification make it possible to achieve residential autonomy also for economically less well-off children. In southern and continental European countries, having highly educated parents increases the likelihood that young adults live on their own and receive financial support from their parents, whereas this likelihood is not graded by level of parental education in Scandinavian countries. Clearly, exiting the parental home is more strongly shaped by the family’s economic resources in continental and southern Europe than in Scandinavian countries.

Research on grandparental care (Bordone, Arpino, & Aassve, 2017) provides another powerful example of policies that enable autonomy in families (defamilialism). In Europe, the likelihood that grandparents provide childcare on a daily basis is strongly linked to the availability of public policy arrangements. In countries where childcare services and parental leaves are most generous, grandparents are least likely to provide daily care to grandchildren while daughters and daughters-in-law are at work. Grandparents are not compelled to step in—because there are public arrangements facilitating the combination of paid work and parenting responsibilities.

Viazzo (2010a, 2010b) has suggested that different explanatory models apply to support transfers in northern and southern Europe. In the northern and western countries with their more generous welfare systems, transfers presumably flow to the neediest, irrespective of any present or future reciprocating help, consistent with the altruistic model. In the southern and eastern countries with their less generous welfare systems, transfers presumably reflect the payment of services and visits and are embedded in current and future obligations of reciprocity (Komter, 2005). Ultimately, intergenerational transfers in southern and eastern Europe would be driven by more morally binding reciprocity obligations (Viazzo, 2010a, 2010b), whereas voluntary obligations (Segalen, 2010) would be more characteristic of intergenerational transfers in Northern and Western Europe. “Voluntary” and “obligatory” seem to contradict one another but match family relations that cherish affection and autonomy (Stuifbergen, Dykstra, & Van Delden, 2010). Viazzo and Segalen did not test their ideas themselves, but recent research shows that the generosity or restrictedness of public provisions variably releases or necessitates normative obligations in interdependent family relationships. Examples follow below.

Leopold and Raab (2011) carried out a fascinating study on cross-national differences in short-term reciprocity, a strategy employed by care recipients to ease the burden of late-life dependency. According to the authors, aging parents strive to maintain balanced exchanges with their adult children to avoid feelings of indebtedness: “They display autonomy by supporting their helping children themselves and thus either repay benefits received or initiate reciprocal support in the short term” (p. 107). Findings show the greatest prevalence of short-term reciprocity in southern European countries where elderly parents depend most strongly on their children’s instrumental support, no prevalence of short-term reciprocity in Nordic countries where family support is complemented by professional care services, and intermediate levels of short-term reciprocity in the continental European countries.

Though Van den Broek and Dykstra (2017) did not employ any direct measure of family obligations, they make a convincing case about the impacts of transfer regimes on adult children’s helping behavior by precluding the possibility of differential selectivity between countries. They show that adult children’s weaker inclination to help frail single-living parents in countries where beds in residential care settings are more widely available is not attributable to “out-selection,” namely that parental needs are less severe in such countries. Neither is the weaker inclination attributable to “in-selection,” namely that adult children and impaired parents are less likely to share a household in such countries. The authors refer to “diffusion of responsibility” to account for adult children’s reluctance to help in countries where beds in residential care settings are more widely available. Knowing that publicly funded care is available seems to undermine adult children’s sense of urgency to step in and provide care to their impaired parents.

Cooney and Dykstra (2011) did not frame their investigation in terms of Viazzo’s morally binding reciprocity obligations and Segalen’s voluntary obligations. Nevertheless, their findings underscore this distinction. They compared intergenerational support patterns in two countries with dramatically different social welfare policy regimes: the Netherlands and the United States of America. Middle-aged adults from the National Survey of Families and Households (NSFH) and the Netherlands Kinship Panel Study (NKPS) reported on financial and instrumental (errands, transportation, household and yard help) support to parents and children. Consistent with their “family-steps-in” hypothesis, the authors find that family obligation norms are stronger predictors of support to parents in the United States than in the Netherlands. Apparently, Americans see it as more critical to act upon shared beliefs about family support given that publicly funded services are not widely available. Living in a welfare regime that offers a relatively high level of support for its citizens seems to allow the Dutch to act on their individual preferences. Similar findings have been reported by Brandt (2013): in SHARE, feelings of obligation are cited most often as reasons for helping family members in southern and continental Europe, whereas the enjoyment of giving is cited most often in northern Europe.

## National Policies Rather Than Transfer Regimes

An issue of debate in the literature is whether regimes, that is clusters of public transfers and services, or specific policies should serve as the basis for explaining cross-national differences in intergenerational transfers in families. For example, Albertini and Kohli (2013) argue that because “regimes can be understood as institutional clusters with a common underlying logic…they should not be dissolved into separate variables” (p. 830). In contrast, Kasza (2002) puts forward that because “most countries practice a disjointed set of welfare policies…policy-specific comparisons may be a more promising avenue for comparative research” (p. 271). Though regimes might be said to provide empirical and theoretical parsimony, the clustering of countries into regime types has limitations, most obviously that national policies within each cluster remain hidden, and that clusters are far from homogeneous (Attias-Donfut et al., 2005; Schenk, Dykstra, & Maas, 2010).

The importance of distinguishing specific policies is evident in recent work on gender inequality. When public care support is offered in cash rather than in kind, the strategy of keeping the money for the family budget and staying at home to provide care is more attractive for women than men, given that men tend to have higher earnings (Javornik, 2014; Lohmann & Zagel, 2016; Saraceno, 2010). Reduced participation in gainful employment contributes to a greater likelihood of late-life poverty among women. Confirming earlier findings, Haberkern, Schmid, and Szydlik (2015) show that women are more likely to provide intensive care to aging parents than men are. However, the gender gap in the provision of such care is highest in countries with low provision of professional home-care services and high public spending on cash benefits. Additional analyses reveal that professional home-care services substitute only for care by daughters, not for care by sons, who show lower levels of engagement generally. Moreover, cash payments encourage intergenerational care but motivate only daughters not sons. The authors conclude that “[a]chieving gender equality in intergenerational care is still a one-way ticket from informal care by women towards State care” (p. 317).

## Cultural Explanations

The question of how much cross-national differences in behavior reflect differences in welfare state constellations and how much they reflect differences in culture is repeatedly addressed in the literature (Pfau-Effinger, 2005). Dominant cultural models of family relations, such as ideas about the gender division of paid and unpaid labor, and ideas about childcare and eldercare responsibilities, differ to a substantial degree across Europe. Traditional family values are characteristic of the Mediterranean countries (Kalmijn & Saraceno, 2008; Marckmann, 2017), though high levels of familialism have also been reported for central and eastern Europe and for Anglo-Saxen countries (Calzada & Brooks, 2013). The models of “proper” family relations underlie welfare state arrangements (Saraceno & Keck, 2010), but Kohli, Albertini, and Haberkern (2010) point out that institutional, structural, and cultural factors do not vary independently among countries; they come in “packages” (p. 241). For that reason, it is difficult to disentangle their effects.

Aassve, Sironi, and Bassi (2013) bring a new perspective to the individualism–familialism divide in Europe, stressing a country’s experience with political independence in the development of liberal attitudes toward the family. They argue that a longer history of self-determination and political autonomy brings greater opportunities to build civic values and social trust. In turn, the higher levels of social trust generate greater confidence in substituting the family’s safety with support found in the wider community. Employing the State Antiquity Index, a measure of the depth of experience with state-level institutions, which correlates highly with social capital (i.e., meaningful contacts outside the immediate family), the authors find the most liberal family attitudes in countries with highest levels of social capital, trust, and voluntary activity. Contrary to popular notions, individualism in the sense of having liberal family attitudes should not be equated with a retreat from engagement in civic and social life. Whereas Reher traced the weak family—strong family divide to religious influences, Aassve and colleagues suggest that it more generally stems from differences in economic and institutional development.

Investigations of intergenerational coresidence have proven to be particularly successful at unraveling cultural and economic factors, although alternative explanations such as the suitability of the housing stock cannot be fully ruled out (e.g., Iacovou, 2010). In a country like Italy, where parents value family togetherness rather than intergenerational independence, the assumption is that parental wealth is devoted to prolonging coresidence. In an elegant natural experiment, Manacorda and Moretti (2006) show that the increase in fathers’ income linked to changes in the Italian Social Security System, resulted in a higher proportion of young men living at home. Apparently, wealthy parents “bribe” their children to remain at home, offering comfort in exchange for their children’s presence. Contrary to the standard explanation that a combination of economic necessity and housing shortages underlies intergenerational coresidence (Newman, 2012; Ruggles, 2007), Manacorda and colleagues show that financial resources enable Italian parents to act on their cultural preferences.

## Wrap-up

Cross-national comparisons constitute a valuable strategy to uncover how macro-level social forces shape intergenerational family relations (Yu, 2015). In this overview, I focused most strongly on research identifying the ways in which public policy arrangements in Europe create and reinforce generational interdependencies in the family realm or—on the contrary—lighten them. The literature provides ample descriptions of the links between public and family streams of support. Generous welfare provisions enable a “specialization” of caring functions, whereby the state performs the demanding tasks requiring professional expertise, and the family provides unstructured and nontechnical help. Generous welfare provisions also enable a “redistribution” of resource flows in families: government spending on older generations encourages transfers from parents to children, whereas government spending on younger generations reduces financial dependency on parents.

Real theoretical progress is visible in three areas of research. These three areas provide inspiring examples for future endeavors. The first area of progress pertains to analyses at the micro level of how family members actually respond to the incentives that different macro contexts provide. The underlying idea is that the generosity or restrictedness of public provisions variably releases or necessitates normative obligations in interdependent family relationships. The second area of progress involves analyses of the incentives for work-family reconciliation linked with specific policies. The package of family policies, for example, pertains to paid and unpaid leaves, daddy quota, targeted and nontargeted cash transfers, and care services, and each has different implications for gender and socioeconomic inequality. The third area of progress is a more nuanced view on the familialism–individualism divide. Rather than fall back on generalized assumptions about enduring cultural norms of intergenerational family solidarity, greater attention is now being paid to how macro-level circumstances impose reliance on family members (familialism) or enable individual autonomy (defamilialism). There is also a greater recognition that individualistic societies tend to have high levels of civic engagement.

A focus on nation states by definition overlooks within-country differences and regional patterns that go beyond national borders. It is important to recognize the limitations of such an approach. Dykstra and Fokkema (2011) find considerable within-country variability in family solidarity patterns, and caution against presuming that countries have a single dominant type of late-life family. Historians have pointed to the persistence of regional differences in family patterns that can be traced to earlier rules of inheritance (Duranton, Rodriguez-Pose, & Sandall, 2009; Mönkediek & Bras, 2014). Perhaps the strongest reason for paying attention to within-country differentiation is decentralization in the public sector. Increasingly, the delivery of health and care services is being delegated to local authorities (Saltman, Bankauskaite, & Vrangbaek, 2007).

Cross-national comparisons of intergenerational family relations not only offer a basis for making theoretical progress but also offer serious methodological challenges (Yu, 2015). There are concerns, for example, about the equivalence of measures across time and country and about the limited number of countries for which comparable and harmonized data sets are available. New methods are being developed to correct for systematic biases induced by unobserved country heterogeneity (e.g., Stegmueller, 2011), and to derive safe statistical inference even with a limited number of countries (e.g., Jackman, 2009).

Another challenge concerns coresidential and noncoresidential households. Paraphrasing Kohli and colleagues (2010), coresidence is the southern and eastern European way of transferring resources from parents to children. Excluding coresidential households from analyses implies that a major means of organizing intergenerational support does not receive the attention it deserves. One option is to apply a Heckman model for selection bias (e.g., Albertini and Kohli, 2013). SHARE does not ask about support exchanges in households, resulting in missing information on a non-negligible number of cases. Leopold and Raab (2011) developed an imputation method using information from parent-child dyads that did not share a household but lived in the same building.

The strong dependency on SHARE data deserves further attention. As Emery and Mudrazija (2015) put forward, strong reliance on this survey and its specific methodological approach may limit the inferences made by researchers examining intergenerational transfers in Europe. They show, for example, that differences in question wording lead to higher reports of financial transfers, particularly among the highly educated, in SHARE than in GGS. Though SHARE is an excellent data source, the authors encourage researchers of intergenerational transfers to validate their findings with multiple data sources.

Considerable progress has been made in reaching an understanding of how and why macro-level factors shape generational interdependence in families. Yet, there are opportunities to improve and expand this scientific body of knowledge. One avenue is to more judiciously theorize about connections between public safety nets (or their absence) and expectations, obligations, rights, and vulnerabilities in the intergenerational family realm. Another avenue involves more critical empirical assessments of theoretical mechanisms. Natural experiments, where changes in types and levels of public provisions might be linked to changes in intergenerational family practices, should be considered more often. Moreover, instead of solely relying on macro-level policy indicators, knowledge about family members’ eligibility to benefits will help clarify patterns of intergenerational assistance.

## Funding

Financial support for work on this chapter comes from the European Research Council Advanced Investigator Grant (ERC, 324211) “Families in Context.”

## Conflict of Interest

None reported.
